# Cardiac complications during the active phase of COVID-19: review of the current evidence

**DOI:** 10.1007/s11739-021-02763-3

**Published:** 2021-05-27

**Authors:** Mohammad Said Ramadan, Lorenzo Bertolino, Tommaso Marrazzo, Maria Teresa Florio, Emanuele Durante-Mangoni, The Monaldi Hospital Cardiovascular Infection Study Group, Emanuele Durante-Mangoni, Domenico Iossa, Lorenzo Bertolino, Maria Paola Ursi, Fabiana D’Amico, Arta Karruli, Mohammad Ramadan, Roberto Andini, Rosa Zampino, Mariano Bernardo, Giuseppe Ruocco, Giovanni Dialetto, Franco Enrico Covino, Sabrina Manduca, Alessandro Della Corte, Marisa De Feo, Stefano De Vivo, Maria Luisa De Rimini, Nicola Galdieri

**Affiliations:** Department of Precision Medicine, University of Campania ‘L. Vanvitelli’ and Unit of Infectious and Transplant Medicine, AORN Ospedali Dei Colli-Monaldi Hospital, Piazzale E. Ruggieri, 80131 Napoli, Italy

**Keywords:** COVID-19, Cardiac complications, SARS-CoV-2, Pathophysiology, Heart disease

## Abstract

Growing reports since the beginning of the pandemic and till date describe increased rates of cardiac complications (CC) in the active phase of coronavirus disease 2019 (COVID-19). CC commonly observed include myocarditis/myocardial injury, arrhythmias and heart failure, with an incidence reaching about a quarter of hospitalized patients in some reports. The increased incidence of CC raise questions about the possible heightened susceptibility of patients with cardiac disease to develop severe COVID-19, and whether the virus itself is involved in the pathogenesis of CC. The wide array of CC seems to stem from multiple mechanisms, including the ability of the virus to directly enter cardiomyocytes, and to indirectly damage the heart through systemic hyperinflammatory and hypercoagulable states, endothelial injury of the coronary arteries and hypoxemia. The induced CC seem to dramatically impact the prognosis of COVID-19, with some studies suggesting over 50% mortality rates with myocardial damage, up from ~ 5% overall mortality of COVID-19 alone. Thus, it is particularly important to investigate the relation between COVID-19 and heart disease, given the major effect on morbidity and mortality, aiming at early detection and improving patient care and outcomes. In this article, we review the growing body of published data on the topic to provide the reader with a comprehensive and robust description of the available evidence and its implication for clinical practice.

## Introduction

Severe acute respiratory syndrome coronavirus 2 (SARS-CoV-2) is a novel beta-coronavirus responsible for the ongoing coronavirus disease 2019 (COVID-19) pandemic, whose real prevalence is thought to be largely higher than reported [1]. Primarily categorized as a respiratory virus, SARS-CoV-2 has also been implicated in a number of extrapulmonary manifestations, including but not limited to renal, neurological and cardiovascular disorders [2]. The relationship between COVID-19 and the cardiovascular system seems quite complex and to-date not completely understood [3].

Full genomic and phylogenetic analysis of SARS-CoV-2 showed it to closely resemble severe acute respiratory syndrome coronavirus (SARS-CoV) (79%), the virus responsible for the first pandemic in the twenty-first century, and distantly the Middle East respiratory syndrome coronavirus (MERS-CoV) (50%), which has caused recurrent outbreaks, most recently on March 17th, 2021 in the United Arab Emirates [4, 5]. The latter viruses are thought to interact with the cardiovascular system, as cardiac diseases (CD) were highly prevalent among infected patients- indicating increased susceptibility of this subgroup, and CD associated with worse outcomes [6, 7].

Alarmingly, CC such as myocardial injury/myocarditis, arrhythmias and heart failure are also increasingly reported in patients with COVID-19, suggesting that COVID-19 could promote CC [8, 9]. Multiple pathophysiological mechanisms have been reported for the latter observation, including systemic cytokine storm, direct myocardial damage, and hypercoagulable state, which are suggested to be induced by SARS-CoV-2 infection [3]. Moreover, there seems to be reciprocated prognostic worsening of both diseases. Despite having low case fatality rates of < 5% in most countries [11], COVID-19 mortality soared in patients with myocardial damage, with some reaching 50% [11]. A recent systematic review and meta-analysis, with more than 20,000 COVID-19 patients, concluded 3 and 11 times higher risk, for both intensive care unit (ICU) admission and ICU mortality, with CD history and acute cardiac injury, respectively, compared to patients with no prior history of CD [12].

Considering these data, there is a stringent need for the Clinician to understand incidence, risk factors, mode of presentation, diagnostic strategies and prognosis of cardiac involvement in COVID-19 and learn how to best prevent or treat it, based on the existing evidence.

In this review, we aim to summarize the available studies investigating cardiac manifestations in adult patients focusing on the acute phase of COVID-19, to increase the understanding of the mutual COVID-heart relationship and highlight the corresponding clinical implications on practice, surveillance programs and guidelines. At variance with previous reports, we exploit in a greater detail the growing body of published data to provide the reader with a comprehensive and robust description of the available evidence and eventually highlight the clinical implications of described data.

## Pathophysiology

**Fig. 1:**
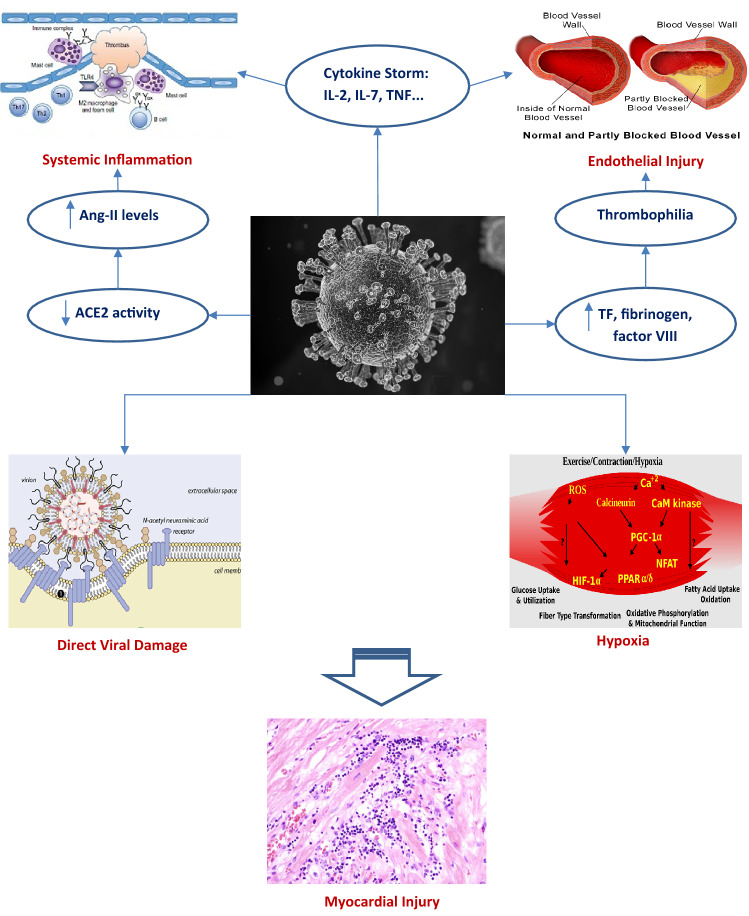
Major pathophysiological pathways linking COVID-19 to heart disease. *Ang-II* Angiontensin-II, *ACE2* angiotensin-converting enzyme 2, *IL* interleukin, *TF* tissue factor, *TNF* tumour necrosis factor. Sources: Sarscov2: "Coronavirus" by Yu. Samoilov is licensed under CC BY 2.0, https://www.flickr.com/photos/yusamoilov/49678500083/in/photostream/. Endothelial Injury: Normal vs. Partially-Blocked Vessel by BruceBlaus, https://commons.wikimedia.org/wiki/File:Blausen_0052_Artery_NormalvPartially-BlockedVessel.png. Inflammation: "Fig. 1 from 'Sex Differences in Inflammation During Atherosclerosis'" by Libertas Academica is licensed under CC BY 2.0, https://search.creativecommons.org/photos/7991b0ee-963d-42bc-bc85-fa2f19396da8. Hypoxia: Exercise/Contraction/Hypoxia, Indolences, https://commons.wikimedia.org/wiki/File:Muscle_pathways.svg. Viral attachment: The coronavirus replication cycle, Crenim at English Wikipedia, edited. https://commons.wikimedia.org/wiki/File:Coronavirus_replication.png

Until our present day, the exact mechanisms of cardiac injury by COVID-19 are not completely understood [3]. The major suggested pathophysiological links between both diseases are described in Fig. 1. One postulated mechanism stems from confirming that SARS-CoV-2, like SARS-CoV, uses its spike (S) protein to attach to the angiotensin-converting enzyme 2 (ACE2) receptor, found on the surface of host cells, and highly expressed in the heart, kidneys, lungs and blood vessels [13]. ACE2 is also part of the renin–angiotensin–aldosterone system (RAAS), whose final product and main effector is Angiotensin-II (Ang-II), a molecule that widely participates in cardiovascular disease (CVD) such as hypertension, MI and heart failure [14]. When Ang-II binds to angiotensin type-1 receptor, it causes vasoconstriction, inflammatory responses, increased blood coagulation, and extracellular matrix remodeling, which explains its strong association with CVD [14]. ACE2 can transform Ang-II into angiotensin-(1–7), which mediates the opposite effects of Ang-II, including vasodilation (also in coronary arteries), decreased proliferation and inflammation and vascular protection [14]. That is why, long before COVID-19, ACE2 was shown to be involved in the pathogenesis of a number of diseases, like diabetes mellitus, heart failure and hypertension, and to play a protective role in the heart and lungs [3, 13]. In patients with SARS-CoV, several studies showed downregulation of ACE2 in the heart and lungs, and reasoned that the same mechanism could explain cardiac injury with COVID-19, as both viruses bind to ACE2 [15]. Albeit the expanding evidence, high ACE2 receptor expression does not necessarily translate into more infection rates nor ACE2 downregulation to cardiac injury [3].

Another suggested mechanism is indirect cardiac injury by stimulating a systemic and dysfunctional immune response, manifesting as a cytokine storm [16]. This was especially seen with severe COVID-19, and displayed as high plasma levels of inflammatory cytokines such as IL-2, IL-7, IL-10, and tumor necrosis factor (TNF) [17]. The increased cytokines in turn mediate local injury to the lung, resulting in diffuse alveolar damage such as hyaline membrane formation and pulmonary edema [18], and can mediate multi-organ failure and myocardial damage. Moreover, bronchoalveolar lavage from patients with severe disease showed increased levels of proinflammatory monocyte-derived macrophages [19]. These activated macrophages secrete a number of molecules, including collagenases, which degrade collagen—a major constituent of fibrous caps—and can lead to plaque rupture, possibly inducing acute coronary syndromes (ACS) [20].

A prothrombotic state was also described in COVID-19, with pulmonary embolism, venous thromboembolism, and disseminated intravascular coagulation (DIC) commonly observed as complications in critically ill patients [3]. In one COVID-19 autopsy series, platelet and megakaryocyte-rich thrombi were found in multiple organs including the heart, kidney and lungs, in some cases despite full anticoagulation [21]. This can result from increased tissue factor, a potent procoagulant from activated macrophages, increased fibrinogen and factor VIII levels and/or from direct endothelial damage that could be caused by SARS-CoV-2 infection [22]. Indeed, in a series of post mortem analysis of COVID-19 patients, SARS-CoV-2 was shown to directly mediate endothelial damage across a number of vascular beds [23]. As endothelial cells are central components to all organs and highly express ACE2, endothelial inflammation induced by SARS-CoV-2 could explain the multiorgan damage in general, and cardiac injury specifically [3].

## Cardiovascular disease and COVID-19

Growing studies highlight the peaking prevalence of comorbid conditions in COVID-19, especially in critical cases [24–26]. Common reported comorbidities included mostly hypertension, followed by diabetes and coronary heart disease [17, 27]. Poor outcomes were associated with these risk factors, including increased hospital stay, admission to the intensive care unit (ICU) and higher morbidity and mortality [24, 25]. In one of the largest cohort studies based in the United Kingdom, factors associated with mortality were studied in adult COVID-19 patients [28]. Obesity (BMI > 40), male sex, diabetes, hypertension, chronic heart disease associated with increased hazard for mortality (2.66, 1.78, 1.58, 1.09 and 1.57, respectively). Similar results were also seen in other studies [24, 25].

**Fig. 2:**
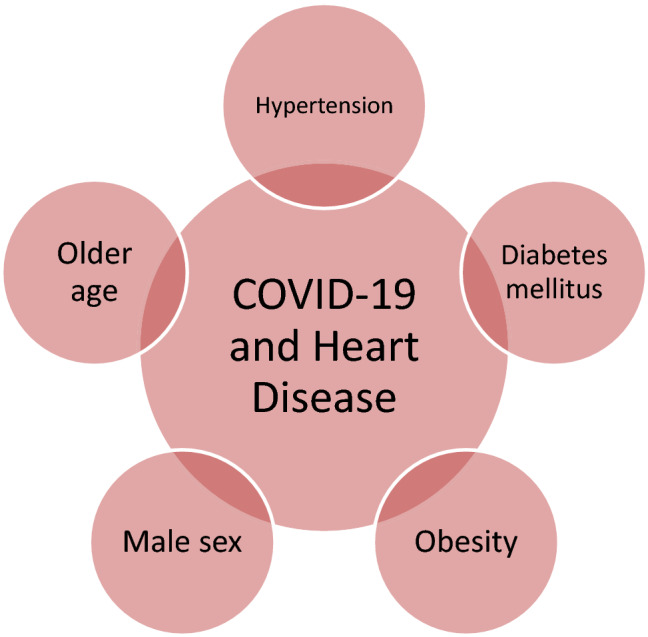
Commonly reported risk factors associated with both heart disease and severe COVID-19

Pathophysiological pathways linking the above risk factors and severe COVID-19 were also assessed. For instance, obesity could aggravate COVID-19 by inducing a pro-inflammatory state along with immune dysfunction, contributed to by the observed leptin and adiponectin deficiency, respectively, and hyperactivation of the complement system—which could lead to a cytokine storm similar to the one described with severe COVID-19 [29]. Hypertension associates with increased RAAS activation, an imbalance between Ang-II and angiotensin-(1–7), and is a major risk for mortality in CVD—all of which could heighten the risk for a severe COVID-19 [30]. Furthermore, immune senescence in the elderly is associated with a low-grade pro-inflammatory state and immune dysfunction, which could delay viral clearance and induce an unregulated inflammatory response and cytokine release, worsening organ damage and delaying recovery [31] (Fig. 2).

## Cardiac complications during acute COVID-19

**Fig. 3:**
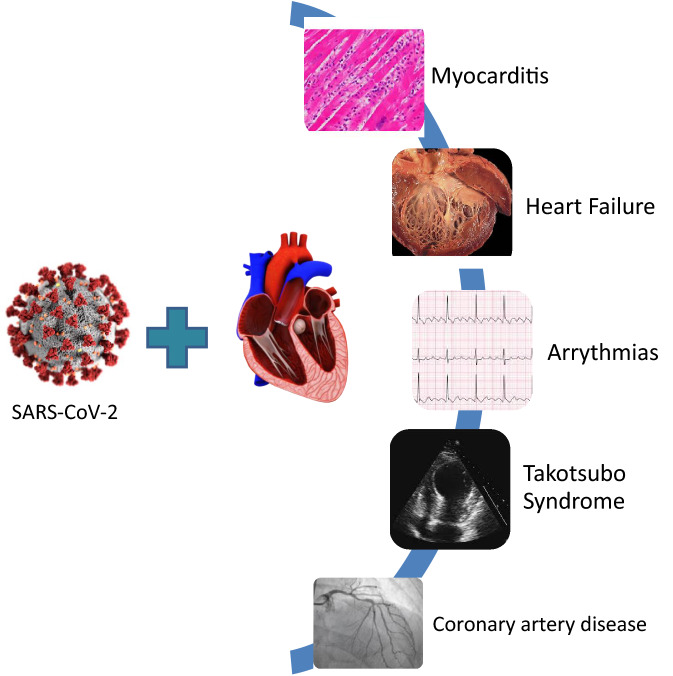
Common cardiac complications associated with COVID-19

Although major COVID-19 complications relate to the respiratory system [12], a wide range of cardiac diseases were proposed to be caused by the disease [8, 32–36], and could be the presenting symptoms of COVID-19, even without the typical respiratory symptoms [35, 37]. The most important cardiac complications that have been described and can be observed during the acute phase of COVID-19 are summarized in a pictorial view in Fig. 3.

### Myocardial injury/Myocarditis

Myocardial injury has been described since early COVID-19 reports from China, where up to 17% of inpatients had elevated troponin, and associated with higher ICU admission (31% vs 4%) and mortality (46% vs 1%) [17, 38]. More recently, a meta-analysis with 26 studies and 11,685 patients found that myocardial injury, assessed mostly by elevated troponin and/or creatinine kinase MB, is present in about 20% of hospitalized patients (range 5–68%) [39]. Despite the increasing reports, pathophysiological mechanisms linking myocardial injury with COVID-19 are still evolving. Some proposed mechanisms include increased systemic inflammation and cytokine storm, respiratory failure and hypoxemia, hypercoagulability leading to increased coronary thrombosis, and direct cardiomyocyte damage through viral attachment [3].

The diagnosis of myocarditis, in general, is one of the most challenging cardiac diagnoses, owing to its wide range of clinical presentations [40]. It can be diagnosed clinically or histologically by endomyocardial biopsy (EMB), the latter being the gold standard, although not commonly performed. The 2013 European Society of Cardiology position statement defines clinically suspected myocarditis in a patient having ≥ 1 clinical criterion such as chest pain or palpitations and ≥ 1 diagnostic criterion such as elevated troponin I or T, electrocardiography (ECG) or cardiac magnetic resonance (CMR) findings [40]. Comparing that with the reported cases of myocarditis in COVID-19 patients, most presented with fever (up to 58%), dyspnea (up to 74%), and chest pain (up to 25%) [41, 42], which coincide with the previous diagnostic symptoms of myocarditis [40]. Moreover, utilized diagnostic methods included cardiac biomarkers levels, ECG, echocardiography, and CMR, showed a wide range of abnormalities reaching up to 90, 84, 79, and 76%, respectively [41, 42], which could further support myocardial injury. CMR changes included late gadolinium enhancement (50%) and diffuse edema and myocardial inflammation (76%); alarmingly, the former could be associated with life-threatening arrythmias and sudden cardiac death [43], and the latter with severe complications including heart failure [44]. Histologic evidence is still limited, EMB, done in a minority of the patients, demonstrated myocardial inflammation with predominant macrophages, confirming myocardial inflammation (eight cases), and one case showed SARS-CoV-2 in cardiomyocytes [41], providing in-vivo proof of possible direct viral injury (Table 1).

**Table 1:** Common signs and symptoms and diagnostic modalities for COVID-19-associated myocardial injury and Takotsubo syndrome

Diagnosis	Signs and symptoms	Echocardiography	ECG	CMR	Biomarkers
Myocardial injury	Dyspnea, fever, chest pain	Systolic/diastolic dysfunction, pericardial effusion	ST-segment elevation and depression, T wave changes, ventricular tachycardia	Increased T1, T2 mapping, late gadolinium enhancement	Elevated
Takotsubo syndrome	Chest pain, dyspnea	Apical akinetic expansion (apical ballooning), hypokinesia hyperdynamic contractility, reduced ejection fraction	ST-segment elevation and/or depression with T-wave inversion	No data available	Elevated

Myocardial injury and myocarditis do not only seem to associate with COVID-19, but to also worsen its prognosis. In an early study from Wuhan, China, with 187 patients, mortality rate increased from 7.62 to 13.3% in those with CVD history, and to 37.5% with both CVD history and acutely increased troponin T [8]. These early observations were also supported by more recent systematic reviews of myocarditis cases, where mortality rates with myocarditis were higher than the reported overall COVID-19 mortality [11], and ranged from 13 to 26% [41, 42, 45]. In addition to increasing mortality, myocardial injury could worsen COVID-19 recovery course. In one study, those with cardiac injury (defined as elevated troponin > 99th percentile) were more likely to require mechanical ventilation (noninvasive: 46.3% vs 3.9%; invasive: 22% vs 4.2%), had more multi-system complications, such as acute respiratory distress syndrome (58.5% vs 14.7%), in addition to higher mortality (51.2% vs 4.5%) [9]. This was also observed in a recent systematic review of cases with myocarditis, where although ~ 80% of inpatients with myocarditis survived, 50% (7/14) needed vasopressors during their hospitalization, 25% (4/14) required inotropic support, and 14% (2/14) required extracorporeal membrane oxygenation [45].

Myocarditis resulting from other viruses, such as Influenza and Epstein-Barr Virus, associated with long-term complications, including heart failure, impaired exercise tolerance, arrythmias and sudden cardiac death [46]. In COVID-19, few studies followed up patients after acute cardiac injury. In a single center pilot study, 48 in-patients were followed up by echocardiography 6 months post-discharge [47]. Results showed that as compared to patients without cardiac injury during hospitalization, those with cardiac injury during the acute COVID-19 phase were more likely to have significant diastolic dysfunction induced by low level exercise [47]. As subclinical myocarditis may carry a high risk for sudden cardiac death during moderate-high intensity physical activity [48, 49], further studies on this specific topic are urgently needed.

Despite suggesting a possible relationship between myocarditis and COVID-19, interpretation of these studies requires caution, as the available evidence remains limited for many reasons [11, 15, 45, 47]. First, few of the reported cases had histologically confirmed myocarditis by EMB—which is still the gold standard for diagnosis; instead, most studies relied on clinical, biomarkers’ levels and imaging criteria for myocardial injury diagnosis [15, 50]. Moreover, the pathophysiological mechanism for such an association is not yet defined, and hence prevention methods could be difficult to predict to reduce the incidence of myocardial injury. Lastly, studies with larger sample sizes and long follow-up could better predict and categorize SARS-CoV-2 associated cardiac injury.

Thus, larger studies with more robust diagnostic techniques and a design including control groups are needed to further assess the relationship between myocarditis and SARS-CoV-2. No further recommendations can be done on myocarditis management in COVID-19, based on existing evidence.

### Takotsubo syndrome

Takotsubo syndrome (TTS) is an acute and transient myocardial contractility impairment leading to acute heart failure, in the absence of coronary artery disease [51]. Several reports described TTS [52–57], more commonly associated with concomitant severe COVID-19 [52–54, 56, 57]. TTS is most often observed in post-menopausal women (~ 90%) [51], which is also seen in COVID-19-associated TTS [50, 52, 54–57], however, TTS is also seen in males [33, 58], and could reach up to 33% of cases [50]. The clinical presentation of TTS could be like that of acute myocardial infarction (MI) with chest pain and/or dyspnea, ST-segment elevation and/or depression with T-wave inversion on ECG, and elevated cardiac biomarkers [51], which closely match the presentations of patients with COVID-19 associated TTS [33, 49, 52–54, 57]. Current literature shows a 4–5% of in-hospital mortality with TTS [51], which could greatly increase with COVID-19, reaching 40% in one series [33].

In addition to possibly increasing mortality, TTS appears to also worsen COVID-19 disease course. Most reported TTS patients had cardiac or respiratory decompensations during their hospitalizations for COVID-19, ranging from increased oxygen requirement, need for vasopressors [33, 50, 52, 53, 55] and in severe cases, ECMO [53, 56]. Although it seems that most patients eventually recover and are discharged, TTS could associate with long-term cardiac and non-cardiac-related morbidity and increased total mortality [51], and so patients with COVID-19 and TTS beyond the acute phase of the disease is warranted.

To date, the exact pathophysiology of TTS is not known. However, major roles of sympathetic stimulation from sudden stress or major physical illness and myocardial inflammation have been proposed [51]. Sympathetic receptors were found to be more concentrated in the cardiac apex, suggesting the apex could be more susceptible for increased levels of catecholamines. Moreover, growing studies show that increased inflammation and particularly ongoing inflammation associates with TTS [36]. In COVID-19, a number of studies showed the pandemic to be associated with significantly increased emotional stress, including anxiety and depression [58], which could increase the risk of TTS by increasing sympathetic stimulation. Furthermore, COVID-19, especially severe cases, are associated with heightened acute and ongoing inflammatory response, which could further increase TTS risk [3].

Thus, current evidence suggests the relationship between TTS and SARS-CoV-2 infection is mediated by emotional stress as well as hyper-inflammatory changes. Overall, the prognosis of TTS in COVID-19 patients seems fair in terms of cardiac functional recovery.

### ACS

The pathophysiological basis of the association between ACS and SARS-CoV-2 stems from the ability of the virus to infect blood vessels in vitro and endothelial cells [23]. It has been demonstrated that SARS-CoV-2 gains entry into cells via ACE2, which is expressed in different organs including lung and heart [3]. Specifically in the heart, ACE2 was shown to be expressed by coronary vascular endothelium, smooth muscle cells, cardiac fibroblasts, epicardial adipocytes, and cardiac myocytes [38]. These associations could explain the increased incidence of multiorgan dysfunction and both micro and macro-vascular thrombotic events in COVID-19 patients [34].

The relationship between COVID-19 and ACS is distinctly controversial. Many studies found decreased incidence of ACS hospitalizations during the pandemic as compared to before it [59]. Whether this was due to reduced incidence or other reasons, including fear of patients of contracting COVID-19 from the healthcare facilities and decreased awareness of ACS, remains unclear. Undoubtedly, COVID-19 posed a challenge for proper ACS diagnosis because of its association with cardiac conditions mimicking ACS, such as myo-pericarditis, coronary artery vasospasm, pulmonary embolism, or stress-induced cardiomyopathy. For instance, in 1 case series of 18 patients with COVID-19 and ST segment elevation on ECG, only 67% of those who underwent angiography had obstructive coronary disease, yet this group had a high mortality rate (72%) [32]. Furthermore, in a retrospective study of 78 patients, higher numbers of complications were reported in ACS COVID-19 patients, as compared to those without COVID-19, including acute respiratory distress syndrome (10%), hemorrhagic stroke (9%), in-hospital mortality (26%) and stent thrombosis [60]. Moreover, this study reported avoidance of PCI as a primary treatment, with more than half of the patients receiving fibrinolytic therapy (59%) rather than the recommended PCI (24%), which coincides with other studies’ conclusions [61].

However, data regarding COVID-19 direct effect on ACS remain conflicting and the association uncertain. For instance, in a recent systematic review of 50,123 patients, the authors concluded no difference emerged in in-hospital mortality and time to obtain medical care in ST-elevation myocardial infarction (STEMI) patients before and during the pandemic. They did report, however, increased door-to balloon-time and declines in STEMI admissions [62]. These studies, despite seemingly with conflicting results, coincide on the significant effect of COVID-19 on ACS diagnosis, prognosis and management; however, the extent of this effect has yet to be determined.

### Arrhythmias

Cardiac rhythm disturbances are among the most common CV complications in the setting of acute COVID-19 [63]. Recent meta-analyses have reported that these events occur in up to 19% of hospitalized patients with SARS-CoV-2 [64] with a substantially higher incidence in those admitted to ICU versus non-ICU encounters (44% vs 6.9%) [65]. However, the real incidence of arrythmias is difficult to establish because of barriers towards the performance of full 12-lead ECG in contagious SARS-CoV-2-infected patients, and thus could be underestimated [63]. Notwithstanding, arrythmias appear to be important to detect because of their major impact on outcome and significant in-hospital mortality risk (RR 7.96 [95% C.I.s 3.77, 16.81], *p* < 0.001; *I*^2^: 71.1%, *p* = 0.02) [64]. Different types of arrythmias have been associated with COVID-19 including atrial fibrillation, atrioventricular blocks, polymorphic ventricular tachycardia, and pulseless electric activity, not always correlating with the severity of lung injury [66]. Supraventricular tachycardias (atrial fibrillation/atrial flutter) are among the most commonly described heart rhythm disturbances with an estimated prevalence of 15.8% (166/1053) as reported by Peltzer et al. [67]. Of note, in this study, 101 out of 166 (60.8%) patients had no prior history of atrial fibrillation or atrial flutter.

A number of mechanisms of cardiac dysrhythmias’ in COVID-19 have been postulated. As mentioned above, SARS-CoV-2 direct damage to cardiomyocytes leading to viral myocarditis could predispose to ventricular arrhythmias [3]. In addition, COVID-19 patients, especially if hospitalized, develop electrolyte disturbances or systemic inflammation [68], which may trigger arrhythmias. Moreover, several drugs administered in the course of the infection may exert arrhythmogenic effects, including azithromycin and hydroxychloroquine [69]. These drugs, inducing QT prolongation, can increase the risk of arrhythmias such as *torsades de point* and ventricular tachycardia. The risk of QT prolongation drastically rises when these drugs are administered in combination regimens. For example, hydroxychloroquine plus azithromycin was found to prolong QT of > 30 ms in 76% of cases [70]. In brief, various arrhythmias frequently occur in COVID-19, correlate with disease severity and worsen prognosis, suggesting close ECG monitoring is warranted in all cases.

### Heart failure

De novo heart failure (HF) has a major impact on prognosis of patients hospitalized for acute COVID-19. Indeed, Zhou et al. found that, among 191 patients, 44 (23%) developed HF with a significant difference between survivors and non-survivors (52% vs 12%, *p* < 0.0001) [38]. Other studies suggest that HF incidence may be even higher. Bieber et al. observed that left ventricular dysfunction (both systolic and/or diastolic) and systolic right ventricular dysfunction occurred in 56% of their total cohort (116 pts) and more frequently in patients with increased troponin (88.8% vs. 14.3%, *p* < 0.001) [71]. Moreover, this complication occurs regardless of a prior history of CD [72], suggesting a direct role of SARS-CoV-2 infection in its pathogenesis.

Heart dysfunction presents with different patterns. Echocardiography performed in the acute stage of the disease reveals that the most common abnormalities are right ventricular (RV) dilation (39%) and left ventricular diastolic dysfunction (16%) [73]. COVID-19 patients are more prone to develop thromboembolic complications, such as venous thrombosis and pulmonary thromboembolism [74], which in turn could underlie RV dysfunction. In addition, alveolar hypoxia in severe cases and acute respiratory distress syndrome (ARDS) in critically ill patients with COVID-19 could worsen RV function by increasing pulmonary vascular resistances and RV afterload [75]. In this context, noninvasive as well as invasive mechanical ventilation may also exert a negative effect [76].

With regards to left ventricular (LV) function, HF with preserved ejection fraction (HFpEF) is a common finding in COVID-19 [73]. This may be due to preexisting subclinical HFpEF or de novo diastolic dysfunction caused by common mechanisms between both diseases like systemic inflammation and hypoxemia [76]. Moreover, HFpEF and COVID-19 seem to share a number of cardiometabolic risk factors including older age and obesity, making it more important to investigate, given the increasing prevalence of obesity in Western countries which could translate into increased susceptibility to severe COVID-19, however, the extent of the effect and the interaction between both diseases has yet to be determined [76]. In summary, published evidence suggests de novo HF frequently complicates COVID-19, implies (RV) overload/dilation and LV diastolic dysfunction, and has a major impact on overall prognosis.

## Conclusions

After more than a year of intense investigations, much has been learned on COVID-19 and CD. SARS-CoV-2 could directly infect the heart via binding to ACE-2 expressed by coronary vascular endothelium, vascular smooth muscle cells, cardiac fibroblasts and myocytes, and epicardial adipocytes. However, direct viral effects are yet to be clearly established.

COVID-19-related myocarditis frequently occurs in conjunction with troponin increase and may have an immune-mediated rather than a viral-induced pathologic mechanism. Its presentation is highly variable and may range from asymptomatic to refractory cardiac dysfunction, largely paralleling the extent of lung involvement. Hyperinflammatory response and some emotional stress underlie the association of COVID-19 with TTS, whose real incidence remains unclear but whose prognosis appears fair. ACS have not been consistently shown to be associated with COVID-19, although health-care disruption due to pandemic has likely exerted an unfavorable effect on their management and prognosis. Of utmost significance, COVID-19 is frequently complicated by arrhythmias, with inflammation, myocarditis and drugs playing the most important pathophysiologic role. Arrhythmias may substantially impact the clinical course of acute COVID-19 and may represent the final cause of cardiac arrest and death. All mentioned cardiac hits may eventually lead to the development of HF with right heart involvement coupled with LV diastolic dysfunction.

In conclusion, an attentive cardiac assessment is warranted in COVID-19 patients irrespective of their prior clinical history of CD. Work up should include ECG recording and, for those hospitalized, serial troponin measurements and careful assessment of fluid and electrolyte balance, with a low threshold for functional echocardiography assessment. This should be of enough quality to analyze RV function and LV diastole. Major uncertainties remain as to whether specific cardiovascular treatments should be avoided or would be beneficial in COVID-19 patients with cardiac involvement. These will likely be the subject of future investigation.

## Data Availability

All data are derived from current literature.
